# A cross-sectional survey of knowledge, attitude and practice associated with COVID-19 among undergraduate students in China

**DOI:** 10.1186/s12889-020-09392-z

**Published:** 2020-08-26

**Authors:** Yaling Peng, Chenchen Pei, Yan Zheng, Juan Wang, Kui Zhang, Zhaohui Zheng, Ping Zhu

**Affiliations:** 1 Department of Clinical Immunology, PLA Specialized Research Institute of Rheumatology & Immunology, Xijing Hospital, Fourth Military Medical University, No. 127 Changle West Rd., Xi’an, 710032 Shaanxi China; 2 Department of Dermatology, Xijing Hospital, Fourth Military Medical University, Xi’an, China

**Keywords:** COVID-19, Knowledge, attitude and practice, Undergraduates

## Abstract

**Background:**

The COVID-19 pandemic has become a great threat to public health, which has greatly impacted the study and life of undergraduate students in China. This study aims to perform a survey of their knowledge, attitude and practice (KAP) associated with COVID-19.

**Methods:**

A cross-sectional survey was designed to gather information regarding the COVID-19 related KAP among undergraduates during the home isolation in the outbreak. Subjects were recruited from 10 universities in Shaanxi Province, China. Enrollees voluntarily submitted their answers to a pre-designed questionnaire online.

**Results:**

A total of 872 subjects (female, 534; male, 338) were enrolled with ages from 17 to 25 years old. This cohort included 430 medical and 442 non-medical students, 580 freshmen and 292 higher school year students. There were 453 from public schools and 442 from private school, residing in 28 regions and provinces at the time of study. Results showed that appropriate knowledge was acquired by 82.34% subjects; the levels were significantly higher in undergraduates from public universities and medical majors than those from private schools and non-medical majors (*p*<0.05). 73.81% subjects reported positive attitudes; females showed significantly higher levels of positive attitudes than males (*p*<0.05). Proactive practice was found in 87.94% subjects. Using a common scoring method, the overall scores for Knowledge, Attitude and Practice were 4.12 ± 0.749 (range: 0 ~ 5), 8.54 ± 1.201 (range: 0 ~ 10), and 8.91 ± 1.431 (range: 0 ~ 10), respectively. There was a positive correlation between attitude and practice (r = 0.319, *p* < 0.05) in the whole study group. Total KAP score was 21.57 ± 2.291 (range: 0 ~ 25), which was significantly different between gender groups and major groups.

**Conclusions:**

Most undergraduates acquired necessary knowledge, positive attitude and proactive practice in response to COVID-19 outbreak; but their KAP scores significantly varied by gender, major and school types.

## Background

In late December 2019, a cluster of patients with an outbreak of pneumonia of unknown cause was reported in Wuhan, China [1]. By January 7, a novel coronavirus, severe acute respiratory syndrome coronavirus 2 (SARS-CoV-2), was identified as the cause to the coronavirus disease 2019 (COVID-19) [2]. Virus quickly spread in other regions in China as well as other countries; human-to-human transmission was proved [3]. World Health Organization (WHO) declared COVID-19 a Public Health Emergency of International Concern on January 30, 2020 [4]. As of July 21, there were totally 14,562,550 confirmed cases and 607,781deaths in the world [5].

To mitigate the outbreak, China quickly announced the highest-level public emergency response and took a series of extraordinary measures during the extended Spring Festival national holiday, including imposing a lockdown in Wuhan. At the same time, a series of other measurements were imposed in the entire country, including rigorous in-door quarantine, person-to-person health check-up, massive disinfection, ubiquitous public health education programs, as well as school and workplace closures [6]. Ubiquitous public health education programs, including internet messages, broadcasting and multimedia reports, virtual classes, e-hospital consultations and flyers of educational materials, played a vital role in public readiness. The outbreak put entire educational system in unprecedented difficult situations; particularly, undergraduate students represented a special group that was at the ages to acquire autonomy and independence of life but with limited experiences. Therefore, their perceptions and behaviors were posited to be greatly affected by the pandemic, which needed to be explored. In this study, we conducted a cross-sectional survey of knowledge, attitude and practice (KAP) associated with COVID-19 among undergraduates in China to evaluate the readiness of Chinese undergraduate students in response to this pandemic.

## Methods

### Study subjects

The enrollees of this survey were from 10 universities (including both public and private ones) in Shaanxi Province, China. Data were collected using an online convenient questionnaire tool, e.g., WJX (https://www.wjx.cn/). The survey was taken from February 23 to 28, 2020, a period when the general public was ordered to stay home isolation at the peak of the outbreak. To ensure the validity and accuracy of collected data, investigators made detailed instructions about the study objectives to all student counselors who distributed the questionnaire to subjects beforehand. The survey tool automatically verified that all questions had to be filled completely before submission and could not be submitted twice. All subjects were informed the survey purpose and signed written consents were obtained online before they started answering the questions (as detailed in Ethics Approval and Consent to Participate Section).

### Survey tool and scoring method

The questionnaire [see Additional File 1] included 7 demographic variables (gender, age, hometown, name of university, type of school, grade, and major) and KAP variables encompassing 5 variables about Knowledge section of COVID-19 (classification of infectious disease, main transmitting route, main clinical manifestation, incubation period and susceptible population), 5 variables about Attitude section towards COVID-19 (human-to-human transmission, wild animal consumption, endurance to emergency, impact on study, and pandemic control measures), and 5 variables of Practice section related to COVID-19 (response to symptoms, frontline rescue help, meeting with cured patients, post-close contact response, and return to school). The Knowledge section was developed with questions from *COVID-19 Diagnosis and Treatment Protocol (Tentative Version Six)* issued by National Health Commission of China [7]. The Attitude and Practice sections were developed by investigators on the basis of the scenarios most likely encountered by undergraduate students.

A common scoring method was used for this KAP questionnaire as follows: (1) 1 point for correct and 0 for incorrect answers in the Knowledge Section, (2) 2 for positive, 1 for neutral and 0 for negative options in the Attitude Section, and (3) 2 for proactive, 1 for neutral and 0 for passive options in the Practice Section. The score ranges were 0 ~ 5 for Knowledge, 0 ~ 10 for Attitude, 0 ~ 10 for practice, and 0 ~ 25 for total KAP.

### Data processing and analysis

Data were analyzed using SPSS 18.0 software. *T test* was used to compare mean values of variables. *Chi square* test was utilized to compare categorical variables and ratios. Pearson or Spearman correlation analysis was used to compare correlations between two variables. *P*<0.05 was considered of statistical significance.

## Results

### Demographic characteristics of subjects

All subjects (*n* = 872) were recruited from 10 universities in Xi’an, Shaanxi Province, including five public and five private schools. Among them, 534 out of 872 (61.2%) were females and 338 out of 872 (38.8%) were males. There were 49.3% (430 out of 872) medical students and 50.7% (442 out of 872) non-medical majors. 51.9% (453 out of 872) attended public schools and 50.7% (442 out of 872) were from private schools. 66.5% (580 out of 872) were in their first year and the rest were in other higher grades. The average age of subjects was 20.1 ± 0.1 with a range between 17 and 25 years old. The hometown of student was reported to cover 28 provinces, autonomous regions and municipalities of China.

### Knowledge of COVID-19

COVID-19 related knowledge was assessed by 5 categories. Each question and answer options were described with graded scores in Table 1. Among the total 4360 collected answers, 3590 (82.3%) were correct. The female students had significantly a higher score for K5 than the males (*p* < 0.05, Table 2); public school students showed significantly higher scores for K1 and K4 than private school students (*p* < 0.05, Table 2). No other statistical significance was found between groups (Table 2).

**Table 1:** COVID-19 knowledge among undergraduate students

Variable categories	Options	Determination/ score	N (%)
**K1:** What type of infectious disease is COVID-19?	• Bacterial.	Incorrect/ 0	17 (2.0)
	• Viral.	Correct/ 1	848 (97.3)
	• I don’t know.	Incorrect/ 0	7 (0.8)
**K2:** What is the main transmission route of COVID-19?	• Respiratory droplets and close contact.	Correct/ 1	862 (98.9)
	• Water.	Incorrect/ 0	2 (0.2)
	• Food.	Incorrect/ 0	5 (0.6)
	• I don’t know.	Incorrect/ 0	3 (0.4)
**K3:** How long is COVID-19 incubation period?	• 1 ~ 14 days.	Correct/ 1	579 (66.4)
	• 3 ~ 7 days.	Incorrect/ 0	21 (2.4)
	• More than 14 days.	Incorrect/ 0	265 (30.4)
	• I don’t know.	Incorrect/ 0	7 (0.8)
**K4:** Who are susceptible to COVID-19?	• The old and young.	Incorrect/ 0	297 (34.1)
	• People are generally susceptible.	Correct/ 1	441 (50.6)
	• Young adults.	Incorrect/ 0	11 (1.3)
	• People with pre-existing diseases.	Incorrect/ 0	117 (13.4)
	• I don’t know.	Incorrect/ 0	6 (0.7)
**K5:** What are the main clinical manifestations of COVID-19?	• Fever and dry cough.	Correct/ 1	860 (98.6)
	• Fatigue.	Incorrect/ 0	3 (0.3)
	• Stuffy and runny nose.	Incorrect/ 0	1 (0.1)
	• Sore throat and myalgia.	Incorrect/ 0	2 (0.2)
	• Diarrhea.	Incorrect/ 0	0 (0.0)
	• I don’t know.	Incorrect/ 0	6 (0.7)

**Table 2:** Comparison of COVID-19 knowledge between different groups

Variables^**a**^	Gender (n, %)				Major (n, %)				Grade (n, %)				Type of school (n, %)			
	Male	Female	χ2	***p***	Medical	Non- medical	χ2	***p***	First year	Other grades	χ2	***p***	Public	Private	χ2	***p***
**K1-correct**	325 (97.0)	523 (97.9)	2.467	0.116	417 (97.0)	431 (97.5)	0.233	0.630	562 (96.7)	286 (98.0)	0.798	0.372	447 (98.7)	401 (95.7)	7.181	0.007
**K2-correct**	334 (98.8)	528 (98.9)	0.007	0.936	427 (99.3)	435 (98.4)	1.509	0.219	574 (99.0)	288 (98.6)	0.193	0.661	449 (99.1)	413 (98.6)	0.579	0.447
**K3-correct**	215 (63.6)	364 (68.2)	1.925	0.165	298 (69.3)	281 (63.6)	3.205	0.073	393 (67.8)	186 (63.7)	1.435	0.231	303 (66.9)	276 (65.9)	0.101	0.751
**K4-correct**	171 (50.6)	270 (50.6)	0.000	0.993	227 (52.8)	214 (48.4)	1.669	0.196	293 (50.5)	148 (50.7)	0.002	0.963	249 (55.0)	192 (45.8)	7.280	0.007
**K5-correct**	330 (97.6)	530 (99.3)	3.992	0.046	426 (99.1)	434 (98.2)	1.243	0.265	573 (98.8)	287 (98.3)	0.366	0.545	446 (98.5)	414 (98.8)	0.199	0.656

^a^ The questions and correct option of each variable are described in Table 1.

Chi-square test was used to compare the percentage of correct knowledge between different groups: male vs. female, medical vs. non-medial majors, first year vs. other grades, public vs. private schools

### Attitude toward COVID-19

Questions about COVID-19 attitude included 5 categories. Each question and answer options were described with graded scores in Table 3. Among all 4360 submitted answers, 3218 (73.81%) showed a positive attitude. The females had significantly higher scores than the males for A1 (*p* < 0.05) and A3 (*p* < 0.05) (Table 4). Students from public schools scored significantly higher than those from private schools for A2 (*p* < 0.05), A3 (*p* < 0.05) and A5 (*p* < 0.05) (Table 4).

**Table 3:** Attitude toward COVID-19 among undergraduates

Variable categories	Options	Determination/ score	N (%)
**A1.** Are you scared by human-to-human transmission of COVID-19?	• No, I’m rational and I can protect myself.	Positive/2	812 (93.1)
	• I don’t care; I feel the same.	Neutral/1	32 (3.7)
	• Yes, I’m panic and don’t know what to do.	Negative/0	28 (3.2)
**A2.** Do you hope the outbreak to stop quickly so you can return to school soon?	• Yes.	Positive/2	673 (77.2)
	• I don’t care.	Neutral/1	147 (16.9)
	• No, I want to stay at home as long as possible.	Negative/0	52 (5.9)
**A3.** What’s your attitude towards wild animal consumption?	• I don’t eat wild animals, and I will accuse consumers.	Positive/2	689 (79.0)
	• I don’t eat personally, but I won’t stop others.	Neutral/1	155 (17.8)
	• I don’t mind having a try.	Negative/0	28 (3.2)
**A4**. Do you think you will be more capable to endure such public health emergence?	• Yes, I’m more educated and thus more capable.	Positive/2	740 (84.9)
	• I will be the same.	Neutral/1	111 (12.7)
	• No, I’m too scared to withstand it anymore.	Negative/0	21 (2.4)
**A5.** Do you think this outbreak has impacted your study?	• Yes, it has.	Negative/0	568 (65.1)
	• No. I’m self-disciplined and my study was not affected at home.	Positive/2	304 (34.9)

**Table 4:** Comparison of COVID-19 attitude between different groups

Variables^**a**^	Gender (n, %)				Major (n, %)				Grade (n, %)				Type of school (n, %)			
	Male	Female	χ2	***p***	Medical	Non- medical	χ2	***p***	First year	Others	χ2	***p***	Public	Private	χ2	***p***
**A1-positive**	307 (90.8)	505 (94.6)	7.911	0.019	401 (93.3)	411 (93.0)	0.601	0.740	543 (93.6)	269 (92.1)	0.841	0.657	419 (94.5)	393 (93.8)	0.579	0.749
**A2-positive**	255 (75.4)	418 (78.2)	5.312	0.070	334 (77.7)	339 (76.7)	5.347	0.069	452 (77.9)	221 (75.7)	5.415	0.067	334 (73.7)	339 (80.9)	6.490	0.039
**A3-positive**	233 (68.9)	456 (85.4)	36.562	< 0.001	353 (82.1)	336 (76.0)	5.101	0.078	471 (81.2)	218 (74.7)	5.044	0.080	370 (81.7)	321 (76.6)	8.819	0.012
**A4-positive**	276 (81.7)	464 (86.9)	4.593	0.101	369 (85.8)	371 (84.0)	0.978	0.613	494 (85.2)	246 (84.3)	0.248	0.883	385 (85.0)	355 (84.7)	3.319	0.190
**A5-positive**	114 (33.7)	190 (35.6)	0.313	0.576	142 (33.0)	162 (36.7)	1.264	0.261	199 (34.3)	105 (36.0)	0.232	0.630	175 (38.6)	129 (30.8)	5.897	0.015

^a^ The questions and positive option of each variable are described in Table 3

Chi-square test was used to compare the percentage of positive attitude between different groups: male vs. female, medical vs. non-medial majors, first year vs. other grades, public vs. private schools

### Practice related to COVID-19

Practice related to COVID-19 was assessed by 5 single-choice questions. Each question and answer option were described with graded scores in Table 5. Among the 4360 answers collected, 3834 (87.9%) indicated a proactive practice. The females had significantly higher score for questions P2 ~ P5, except P1 (Table 6). Students in higher grades had a significantly higher score for P5 than freshmen (Table 6).

**Table 5:** Practices related to COVID-19 among undergraduates

Variable categories	Options	Determination/ score	N (%)
**P1:** What would you do if you had fever and dry cough?	• I will analyze the situation rationally. Stay home for observation and self-quarantine or go to a hospital for a treatment.	Proactive/2	817 (93.7)
	• I want to go to a hospital, but I’m afraid to be infected.	Neutral /1	39 (4.5)
	• I feel panic and don’t know what to do.	Passive/0	16 (1.8)
**P2:** If the country needs you, are you willing to help the frontline rescue?	• Yes, every citizen shall bear the country’s burden.	Proactive/2	812 (93.1)
	• I’m not sure and need suggestions from the family.	Neutral /1	45 (5.1)
	• No, it’s too dangerous.	Passive/0	15 (1.7)
**P3:** What would you do if you had close contact with confirmed cases?	• Proactively report to the community and stay home in quarantine as required.	Proactive/2	852 (97.7)
	• Same as before.	Neutral /1	3 (0.3)
	• I feel panic and don’t know what to do.	Passive/0	17 (2.0)
**P4:** What would you do if someone cured from COVID-19 wanted to meet you?	• I will meet them and show more kindness.	Proactive/2	697 (79.9)
	• I will meet them just like before.	Neutral /1	40 (4.6)
	• I’ll find an excuse to keep away from them.	Passive/0	135 (15.5)
**P5:** What will be your top priority when the pandemic ends?	• I will go back to school and restart normal study.	Proactive/2	656 (75.2)
	• Same as before.	Neutral /1	3(0.3)
	• The outbreak is too scary. I need to enjoy my life as much as possible.	Passive/0	213(24.4)

**Table 6:** Comparison of COVID-19 practice between different groups

Variables^**a**^	Gender (n, %)				Major (n, %)				Grade (n, %)				Type of school (n, %)			
	Male	Female	χ2	***p***	Medical	Non-medical	χ2	***p***	First year	Others	χ2	***p***	Public	Private	χ2	***p***
**P1-proactive**	311(92.0)	506(94.8)	3.126	0.210	411(95.6)	406(91.9)	5.219	0.074	550(94.8)	267(91.4)	3.985	0.136	419(92.5)	398(95.0)	2.295	0.318
**P2- proactive**	297(87.9)	515(96.4)	24.885	0.000	407(94.7)	405(91.6)	4.196	0.123	542(93.5)	270(92.5)	1.191	0.551	420(92.7)	392(93.6)	1.331	0.514
**P3- proactive**	326(96.5)	526(98.5)	3.890	0.049	421(97.9)	431(97.5)	0.152	0.696	565(97.4)	287(98.3)	0.662	0.416	443(97.8)	409(97.6)	0.031	0.860
**P4- proactive**	273(80.8)	424(79.4)	9.294	0.010	348(80.9)	349(79.0)	1.903	0.386	470(81.0)	227(77.7)	1.504	0.471	367(81.0)	330(78.8)	0.747	0.688
**P5- proactive**	270(79.9)	386(72.3)	6.411	0.011	324(75.4)	332(75.1)	0.006	0.936	421(72.6)	235(80.5)	6.494	0.011	338(74.6)	318(75.9)	0.192	0.661

^**a**^ The questions and proactive option of each variable are described in Table 5.

Chi-square test was used to compare the percentage of proactive practice between different groups: male vs. female, medical vs. non-medial majors, first year vs. other grades, public vs. private schools

### Comparison of COVID-19 related KAP scores between different groups

In the whole study population, the overall score for Knowledge was 4.1 ± 0.7 (range: 0 ~ 5), Attitude was 8.5 ± 1.2 (range: 0 ~ 10), and Practice was 8.9 ± 1.4 (range: 0 ~ 10). There was a positive correlation between Attitude and Practice scores (r = 0.319, *p* < 0.05). Subgroup analysis indicated that Knowledge level was significantly higher in students from public schools and medical programs than those from private schools and non-medical majors (*p*<0.05) (Table 7). There was a significantly higher score of Attitude in females than males (*p*<0.05) (Table 7). Practice level was found no statistically significant difference between groups by gender, grade, major or school style (Table 7).

**Table 7:** Comparison of COVID-19 related KAP scores between different groups

	Variables	(n)	Knowledge		Attitude				Practice			Total KAP		
			$\overline{x}\pm s$	*t*	*p*	$\overline{x}\pm s$	*t*	*p*	$\overline{x}\pm s$	*t*	*p*	$\overline{x}\pm s$	*t*	*p*
**Gender**	Male	338	4.07 ± 0.78	−1.537	0.125	8.30 ± 1.33	−4.706	0.000	8.92 ± 1.46	0.176	0.860	20.55 ± 2.38	−2.827	0.005
	Female	534	4.15 ± 0.73			8.69 ± 1.09			8.90 ± 1.41			20.98 ± 2.04		
**Major**	Medical	430	4.17 ± 0.71	2.240	0.025	8.59 ± 1.16	1.058	0.290	9.00 ± 1.37	1.751	0.080	21.00 ± 2.13	2.437	0.015
	Non-medical	442	4.06 ± 0.78			8.50 ± 1.24			8.83 ± 1.49			20.64 ± 2.23		
**Grade**	First year	580	4.13 ± 0.73	0.686	0.493	8.59 ± 1.16	1.758	0.079	8.89 ± 1.43	−0.474	0.636	20.85 ± 2.17	0.588	0.557
	Others	292	4.09 ± 0.78			8.44 ± 1.28			8.94 ± 1.43			20.75 ± 2.23		
**School type**	Public	453	4.18 ± 0.75	2.636	0.009	8.52 ± 1.25	−0.548	0.584	8.89 ± 1.51	−0.472	0.637	20.82 ± 2.29	0.082	0.935
	Private	419	4.05 ± 0.74			8.57 ± 1.15			8.93 ± 1.35			20.81 ± 2.07		

Independent sample T-test was used to compare the scores of KAP between different groups: male vs. female, medical vs. non-medial majors, first year vs. other grades, public vs. private schools

Total KAP score was 21.6 ± 2.3 among the subjects, in which a positive correlation was observed between the Attitude and Practice (r = 0.319, *p* < 0.05). Total KAP score was significantly different among gender groups and major groups(*p* < 0.05, *p* < 0.05) (Table 7).

## Discussion

Since the outbreak in epicenter Wuhan in December 2019, COVID-19 has rapidly become a threat to global public health and led to substantial socioeconomic damages in the whole world. Vigorous measurements have been enforcedly implemented including lockdown of Wuhan and community quarantine by Chinese central and local governments since the outbreak to mitigate the disease effectively. In addition, public health education has been recognized as an effective measure to prevent and control public health emergency for the public preparedness against such situation. It will lead the public to acquire appropriate knowledge, mitigate panic and seek for positive attitude, and comply with aligned and desired practices. All these KAP elements have been considered crucial to ensure effective prevention and control of the pandemic.

This cross-sectional survey on 872 undergraduate students found that most of them were well informed with COVID-19 related knowledge, showed positive attitude and proactive practice during the outbreak, indicating that effective health education was delivered by the massive public education campaigns (especially via Internet). This result is consistent with many other reports on H1N1 related KAP among university students in South Korea, UK and Hong Kong [8–10]. Our study also revealed that females had significantly higher score on the knowledge of “main clinical manifestation of COVID-19”, in line with the result of an investigation on MERS in Saudi Arabian [11]. It has been shown that women is superior to men in terms of the knowledge and practice (hand hygiene, wearing a mask) related to infectious diseases (eg: H1N1, SARS, and MERS, *etc*) [10–15]. This gender difference also has been shown in our study that are evidenced by a better score on attitude score and KAP total score for females than males, including proper and rational protective measurements to reduce the risk of human-to-human transmission and aversion to wild animal consumption.

Students from public schools and medical programs showed a higher score for COVID-19 related knowledge. This could be explained by the characteristic educational situations in China. Since the past two decades, private universities have been begun to be established as a role player supplementary to the public educational system as per governmental policies. Comparing to private schools, public universities have been innately superior in their numbers and scales, quantity and quality of students and teachers, as well as supports from the authorities, etc [16]. Medical students showed a good score of knowledge which could be explained by their trainings in clinical medicine and public health. Their obligations and responsibilities to fight against this pandemic as future medical professionals are thought to drive them to present more positive attitudes and proactive practices during this public health emergency [17].

This investigation has some limitations. First, the convenient sampling method could not avoid the bias of subjective selection, thus diminishing the internal validity. Second, the nature of cross-sectional study design was unable to determine causality between the variables. Third, our subjects were enrolled from ten universities in a single city, though they were staying at home for isolation in twenty-eight provinces and regions across the country at the time of survey. This might not reflect the actual situation of undergraduate students in Chinese universities as a whole.

To our knowledge, this study is the first investigation on the current KAP related to COVID-19 among Chinese undergraduate students. Therefore, it provides valuable insights into public health education and preventative measures in Chinese universities during the COVID-19 pandemic. Our results indicate that the majority of Chinese undergraduate students have acquired the basic knowledge of COVID-19, but their performance may vary by school types and majors. Attitude towards COVID-19 shows a gender disparity. Taken together, these results suggest that gender, major and school styles potentially affect student’s responses to COVID-19 outbreak and acquisition of public health education, which should be drawn awareness to education and health authorities. These factors should also be accounted to formulate contingency plans or trainings for the students against similar public health emergencies in future.

## Conclusion

Most Chinese undergraduate students understood the basic information, possessed positive attitude and presented proactive practice towards the outbreak of COVID-19, indicating the efficacy and success of present public health campaigns. However, results also revealed that gender, major and school types should be taken into consideration when health and education authorities tailor public health trainings and improve their preventative measures against this epidemic.

## Supplementary information

**Additional file 1.** English translation of the questionnaire.

## Data Availability

The datasets used and/or analyzed during the current study are available from the corresponding author on reasonable request.

## Abbreviations

KAP: Knowledge, attitude and practice
COVID-19: Coronavirus disease 2019
SARS-CoV-2: Severe acute respiratory syndrome coronavirus 2
WHO: World Health Organization
SARS: Severe acute respiratory syndrome
MERS: Middle east respiratory syndrome
