# Supplementary material for: A cross-sectional survey of knowledge, attitude and practice associated with COVID-19 among undergraduate students in China
Source: BMC Public Health. 2020 Aug 26;20:1292. doi: 10.1186/s12889-020-09392-z (PMC7447607; doi:10.1186/s12889-020-09392-z)
Supplement: Supplementary file 1 — Additional file 1. English translation of the questionnaire. [file 12889_2020_9392_MOESM1_ESM.docx]

***Additional file 1 (English translation):***

**Questionnaire for Survey of Knowledge, Attitude and Practice Associated with COVID-19 among Undergraduate Students**

*indicates a single choice question.

**Personal Information**

**1. Gender (*):** Male Female

**2. Age:**  years old

**3. Hometown:**

**4. Name of university:**

**5. Type of university (*)** Public school Private school

**6. Grade:** Year 1 Year 2 Year 3 Year 4 Year 5

**7. Major (*):** Medical Non-medical

**8. What type of infectious disease is COVID-19? (*)**

Bacterial Viral I don’t know.

**9. What is the main transmission route of COVID-19? (*)**

Respiratory droplets and close contact. Water. Food. I don’t know.

**10. How long is COVID-19 incubation period? (*)**

1~14 days. 3~7 days. More than 14 days. I don’t know.

**11. Who are susceptible to COVID-19?**

The old and young. People are generally susceptible. Young adults.
 People with pre-existing diseases. I don’t know.

**12. What are the main clinical manifestations of COVID-19? (*)**

Fever and dry cough. Fatigue. Stuffy and runny nose. Sore throat and myalgia. Diarrhea. I don’t know.

**13. Are you scared by human-to-human transmission of COVID-19? (*)**

No, I’m rational and I can protect myself. I don’t care; I feel the same. Yes, I’m panic and don’t know what to do.

**14. Do you hope the outbreak to stop quickly so you can return to school soon? (*)**

Yes. I don’t care. No, I want to stay at home as long as possible.

**15. What’s your attitude towards wild animal consumption? (*)**

I don’t eat wild animals, and I will accuse consumers. I don’t eat personally, but I won’t stop others. I don’t mind having a try.

**16. Do you think you will be more capable to endure such public health emergence? (*)**

Yes, I’m more educated and thus more capable. I will be the same. No, I’m too scared to withstand it anymore.

**17. Do you think this outbreak has impacted your study? (*)**

Yes, it has. No. I’m self-disciplined and my study was not affected at home.

**18. What would you do if you had fever and dry cough?**

I will analyze the situation rationally. Stay home for observation and self-quarantine or go to a hospital for a treatment.

I want to go to a hospital, but I’m afraid to be infected.

I feel panic. I don’t know what to do.

**19. If the country needs you, are you willing to help the frontline rescue?**

Yes, every citizen shall bear the country’s burden.

I’m not sure and need suggestions from the family.

No, it’s too dangerous.

**20.What would you do if you had close contact with confirmed cases?**

Proactively report to the community and stay home in quarantine as required.

Same as before.

I feel panic and don’t know what to do.

**21.What would you do if someone cured from COVID-19 wanted to meet you?**

I will meet them and show more kindness.

I will meet them just like before.

I’ll find an excuse to keep away from them.

**22. What will be your top priority when the pandemic ends?**

I will go back to school and restart a normal study.

Same as before.

The outbreak is too scary. I need to enjoy my life as much as possible.
